# Clinical effectiveness of a digitally delivered balance program for older adults: a nonrandomized controlled trial

**DOI:** 10.57264/cer-2026-0070

**Published:** 2026-07-21

**Authors:** Folasade Phillips, Sandhya Yadav, Cynthia Castro Sweet, Rachel NS Foster Kirk, Mindy Hong, Samuel Mink, Jeffrey Krauss

**Affiliations:** 1 Hinge Health, Inc., 455 Market Street, Suite 700, San Francisco, CA 94105, USA; 2 Midi Health, Inc., 195 Page Mill Road, Suite 103, Palo Alto, CA 94306, USA; 3 Bonafide Health, LLC. 500 Mamaroneck Avenue, Suite 510, Harrison, NY 10528, USA; 4 Teladoc Health, Inc., 2 Manhattanvillle Road, Purchase, NY 10577, USA; 5 Stanford University School of Medicine, Division of Physical Medicine & Rehabilitation, 450 Broadway Street, Redwood City, CA 94063, USA

**Keywords:** digital health, exercise therapy, fall prevention, healthcare utilization, older adults, telerehabilitation

## Abstract

**Aim:**

To evaluate the clinical effectiveness of a digitally delivered balance program relative to an attention-control comparison group in older adults at risk for falls.

**Materials & methods:**

This nonrandomized controlled trial recruited adults aged 65 years and older with moderate-to-high fall risk. Participants were assigned to a digital balance program (exercise therapy, education and health coaching) or an attention-control group (education materials). Outcomes were assessed via self-report surveys at baseline and 3 months. Analyses including all assigned participants evaluated changes in fall rate, fall severity, physical function and medical care utilization.

**Results:**

A total of 687 participants were included in the analysis (intervention: n = 344; attention-control: n = 343). The mean age was 68.8 years, and 74.1% of participants were female. In the primary analysis, adjusting for baseline factors, the intervention group demonstrated a 37% lower fall rate compared with the attention-control group at 3 months (IRR: 0.63, 95% CI: 0.48–0.82, p < 0.001). The intervention group also demonstrated significant improvement in physical functioning (β = 6.86, 95% CI: 3.80–9.92, p < 0.001) and lower odds of emergency department visits (OR: 0.43, 95% CI: 0.24–0.76, p = 0.004). Intervention participants engaged in an average of 25.2 exercise therapy sessions over the 12-week period.

**Conclusion:**

Findings suggest that participation in the digital balance program was associated with reductions in self-reported fall rates, improvements in self-reported physical function, and lower odds of emergency department utilization. High engagement levels further indicate that digitally delivered programs offer a viable option for improving health outcomes in this population.

**Trial Registration:**

Clinicaltrials.gov NCT06868680 retrospectively registered 6 March 2025.

## Plain language summary: Can a digital exercise program help prevent falls in older adults?

**What is this article about?:**

Falls are a major cause of injury for adults aged 65 and older. While exercise can help prevent falls, many older adults find it difficult to attend in-person classes due to transportation or mobility issues. This study looked at whether a digital balance program (delivered through an app on a smartphone or tablet) could help reduce falls. The program included exercise therapy, education and support from a health coach and physical therapist. Researchers compared 344 participants who used the app to 343 participants who received only educational emails to see if the app helped reduce falls and improve overall health.

**What were the results?:**

After 3 months, participants using the digital program reported significantly fewer self-reported falls compared with the group that only received educational emails. As part of an exploratory analysis, participants using the app also reported significant improvements in their physical ability to move and do daily tasks. In another exploratory finding, those in the digital program group reported fewer emergency department visits during the study. Participants were highly engaged with the app, completing an average of two exercise sessions per week.

**What do the results mean?:**

These findings suggest that digital exercise programs can be an effective and accessible way for older adults with baseline digital literacy and device access to improve their balance and physical health from home. By reducing patient-reported fall risks and emergency hospital visits, such programs may help tech-proficient older adults live independently for longer while reducing the burden on the healthcare system.

Falls are a leading cause of injury-related death and increased healthcare utilization among adults aged 65 years and older, accounting for over 38,000 deaths and millions of emergency room visits annually in the US [1–3]. Beyond the estimated $50 billion in annual economic burden [4], falls contribute to functional decline, social isolation, and a cyclical fear of falling that further increases risk [5–7]. While the US Preventive Services Task Force (USPSTF) recommends exercise interventions to prevent falls [8] and traditional programs can reduce fall rates by up to 23% [9], barriers such as transportation and mobility limitations often hinder participation in facility-based programs [10]. Digitally delivered balance training offers a potential solution to these barriers, with emerging evidence suggesting high adherence and efficacy [11,12]. To advance beyond historical fall prevention programs, the digital program evaluated in this study uniquely integrates personalized exercise therapy, dual-task training and educational modules with a dedicated healthcare team to optimize behavioral adherence. This study evaluated the initial clinical effectiveness of a digitally delivered balance program for reducing falls, improving physical function and decreasing healthcare utilization among older adults at risk for falls.

## Materials & methods

### Study design

A nonrandomized controlled trial was conducted to compare early clinical outcomes among adults aged 65 years and older who participated in a digitally delivered balance program, versus those assigned to an attention-control group. Study outcomes were assessed over a 3-month study period.

### The balance program

**Figure 1.**
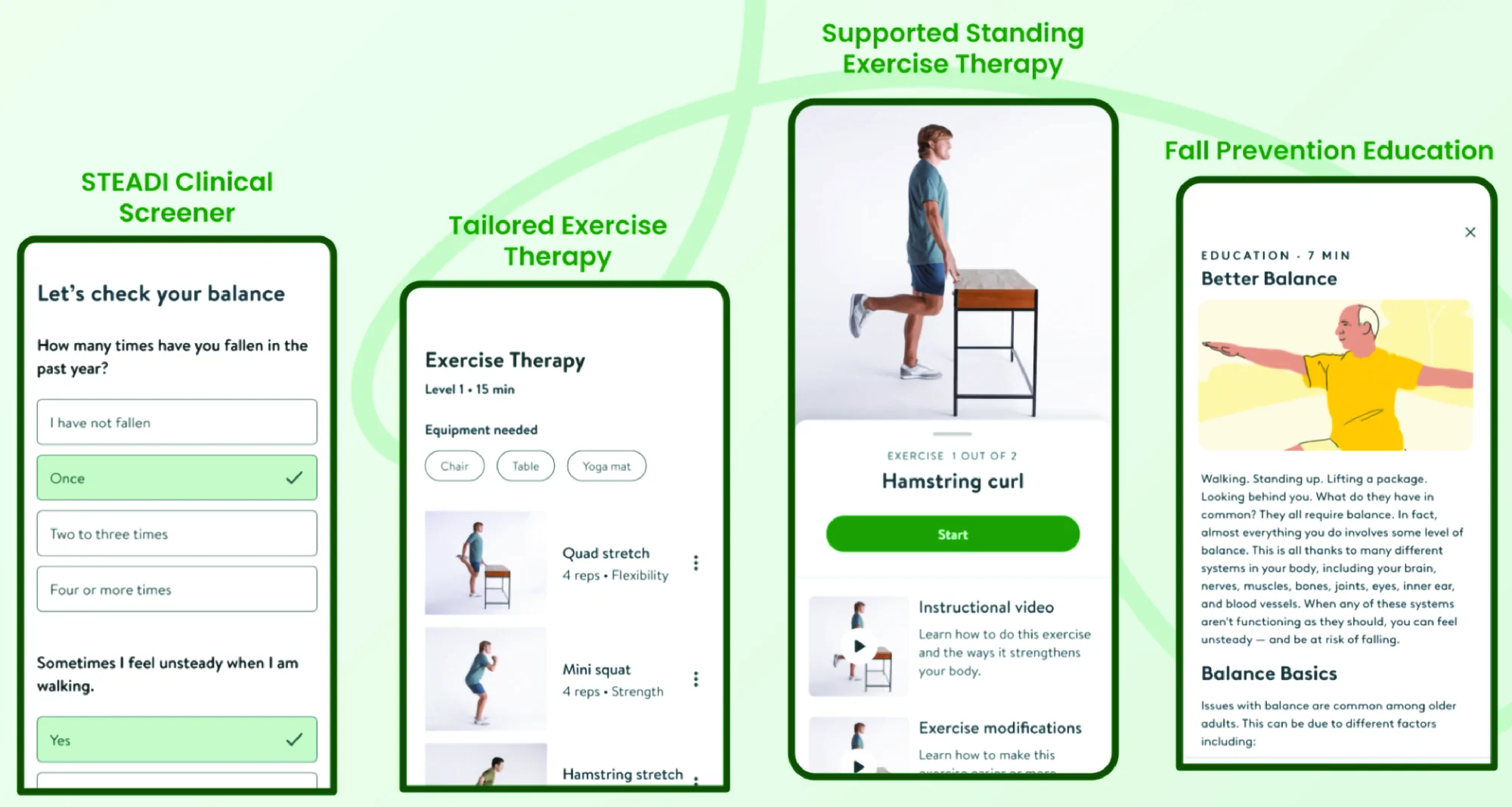
Representative user interface screens of the digital balance program.

Participants in the intervention group received a digitally-delivered program designed to improve balance, strength and mobility through exercise therapy, education, and health coaching (Hinge Health, Inc., CA, USA). The core component is a structured exercise therapy regimen accessed via a smartphone or tablet app (Figure 1). Exercises were prescribed and personalized by a physical therapist across three core clinical pillars, based on initial video assessments and ongoing progress. Lower-body balance and strength training was stratified into four streams according to a participant’s functional ability: seated (chair-based mobility), supported standing (using a wall or rail to maintain stability), unsupported standing (independent upright stance) and floor exercises (for fall-recovery transitions). Low-impact flexibility movements targeted joint range of motion. Dual-task training involved simultaneous physical balance and cognitive challenges to train motor coordination under real-world conditions.

Participants were encouraged to complete 10–15 minute sessions at least three-times per week, with frequency and intensity individualized based on baseline assessments. The program also included digital educational modules related to fall prevention, home safety and strategies to improve daily function. A dedicated healthcare team composed of a certified health coach and a licensed physical therapist provided remote behavioral support, progress monitoring and personalized exercise adjustments via in-app messaging and video visits. Participants received automated reminders and engagement prompts from the app to encourage ongoing adherence.

Regular remote monitoring enabled the healthcare team to identify and address safety concerns or barriers to engagement throughout the 12-week intervention period.

### Attention-control group

Participants assigned to the attention-control group received educational materials on fall prevention via email on a bi-weekly cadence, totaling seven articles over the 12-week study period, matching the education content provided to the intervention group, with no secondary delivery or reading reminders provided. Topics included balance basics, fall risk factors, home safety and strategies for physical and psychological self-efficacy. Content was based on evidenced-based guidelines from the CDC, the STEADI (Stopping Elderly Accidents, Deaths and Injuries) initiative, and medical research on vestibular function and cognitive health.

### Procedures

Recruitment was managed centrally by a national consumer marketing research panel, which deployed two distinct, IRB-approved advertisements concurrently, each framed as a separate study: one for the digital balance program and one for the attention-control group. Prospective participants self-selected into a study arm based on the advertisement they responded to, meaning group allocation was determined prior to eligibility screening, and no randomization was utilized. Inclusion criteria required individuals to be aged 65 years or older, have smartphone access or be willing to use a program-provided tablet with wifi access, and have a STEADI score of 4 or greater, indicating moderate-to-high fall risk [13]. Participant recruitment and data collection for this study were conducted from February 2024 through August 2024. Exclusion criteria comprised a STEADI score <4, recent musculoskeletal surgery (<3 months), cognitive or neurological disorders that would impede protocol compliance (e.g., dementia, Parkinson’s, history of stroke), or any medical contraindication to light physical activity. Eligible participants were then enrolled into parallel cohorts. This concurrent allocation strategy was used to minimize temporal biases typical of nonrandomized designs, and the inherent self-selection mechanism was addressed analytically by adjusting all final inferential models for baseline clinical and demographic covariates. Both groups completed online surveys at baseline and 3 months to assess falls and lifestyle factors. Participation was voluntary, confidential and compensated with electronic gift cards. All procedures were approved by the WIRB-Copernicus Group (WCG) Institutional Review Board.

### Study outcomes & measurements

Baseline demographics including age, sex, race, ethnicity, education level, marital and employment status, along with self-reported health status (measured using the standard 5-point scale: Excellent, Very Good, Good, Fair, Poor), were captured via an online survey completed by all participants at study entry.

The primary outcome was fall incidence rate, defined as the annualized number of falls per participant based on self-report responses to the question: “How many times have you fallen in the past [1 year/3 months]?” At baseline, annualized fall rate was calculated by dividing the total number of falls reported in the prior 12 months by 12. At 3 months, annualized fall rate was calculated by dividing the total number of falls reported between baseline and month 3 by 0.25 person-years [9,14]. Incidence rate ratios (IRR) for fall rate at month 3 were calculated between groups, adjusting for baseline covariates.

Fall severity was assessed with a single question: “Based on the following categories, how severe was the worst fall you experienced?” during the measurement period. Severity categories were based on the National Database of Nursing Quality Indicators (NDNIQ) standards [15].

- None: No injuries (no signs or symptoms) resulting from the fall.
- Minor: Resulted in application of a dressing, ice, cleaning of a wound, limb elevation, topical medication, bruise or abrasion.
- Moderate: Resulted in suturing, application of steri-strips/skin glue, splinting, or muscle/joint strain.
- Major: Resulted in surgery, casting, traction, required consultation for neurological, internal injury or receiving blood products for coagulopathy as a result of the fall.

As an exploratory outcome, physical function was measured using the Physical Functioning Scale (PFS) of the ShortForm-36, which evaluates capabilities and limitations in activities of daily living and physical activity [16]. Scores are standardized on a 0–100 scale, where higher scores indicate better physical functioning and fewer limitations.

A second exploratory outcome was change in self-reported healthcare utilization in the past 30 days, a timeframe selected to minimize recall bias in an older population while capturing active utilization rates at 3 months. Participants reported healthcare encounters over the past 30 days across specific categories: visits to a primary care provider, physical therapist, orthopedic surgeon; diagnostic imaging; injections; emergency department (ED) visits; overnight hospital stays; and surgeries.

### Additional measurements

All participants were screened at baseline using the Stopping Elderly Accidents, Deaths, and Injuries (STEADI) questionnaire. Higher scores indicate greater risk for falling. The seven-item Short Falls Efficacy Scale-International (Short FES-I) was administered at baseline to assess fear of falling, with higher scores indicating greater concern [17].

Among intervention group participants, engagement metrics were tracked throughout the 3-month period, including the total number of exercise therapy sessions completed, educational articles read within the application, and messages sent to the care team. Participant engagement data were completely objective. Completed exercise therapy sessions, messages sent, and articles read were natively logged and tracked via the application. For the analysis of engagement data, these metrics were pulled directly from the backend for each participant, providing a precise audit of program exposure free from self-report or recall bias.

### Adverse event & data safety monitoring

Participant safety and data integrity were governed by an IRB-approved Data Safety Monitoring Plan, with study investigators continuously evaluating study data. Under this, participants were instructed to report any program-related adverse events to their care team, which were logged subject to Western Institutional Review Board (WCG) reporting guidelines.

### Statistical analysis

All analyses were conducted using R statistical software (version 4.0.5; R Foundation for Statistical Computing). Data were collected at baseline (study enrollment) and month 3 via self-report surveys administered through the Qualtrics online survey platform. Participants received up to three email reminders to encourage survey completion at each time point.

Sample size was calculated to detect a 35% reduction in the fall rate (IRR of 0.65) between groups, with 80% statistical power at a 5% significance level [18]. This required an analytic sample of 378 participants (189 per group). To account for an anticipated 45% attrition rate, a conservative estimate reflecting the pragmatic, real-world nature of this remote digital health study, the recruitment target was set at 687 participants (344 per group). This ensured the final sample would remain sufficiently powered despite potential loss to follow-up.

Primary efficacy analyses were performed on all assigned participants, regardless of program adherence or study completion. Descriptive statistics were generated for all demographic and baseline variables, with means and standard deviations summarized for continuous variables and frequencies and percentages for categorical variables. Between-group comparisons at baseline were assessed using two-tailed *t-*tests for continuous variables, and either chi-squared tests or Fisher’s exact tests for categorical variables (Fisher’s exact test was applied when expected cell counts were below five).

Missing outcome data (n = 88 participants with no month 3 outcome data) were addressed using Multiple Imputation by Chained Equations utilizing Predictive Mean Matching. The imputation model included baseline demographics, comorbidities and baseline outcome scores as predictors; baseline STEADI and fall severity were excluded due to excessive missingness. Five imputed data sets (m = 5, 10 iterations) were generated with convergence verified via trace plots. To address missing outcome data at month 3, we used multiple imputation. For the primary outcome of fall incidence rate at 3 months, negative binomial regression was fitted to the full analytic sample, adjusting for baseline covariates. Ordinal logistic regression was applied to compare fall severity categories between groups. Changes in physical function scores over time were assessed using mixed-effects linear regression models, both for the overall sample and for subgroups with low baseline physical function (PFS score <50).

A multivariable logistic regression model was employed to evaluate the association between participation in the balance program and the likelihood of change in healthcare utilization. To assess the robustness of our primary findings against differential attrition and potential departures from the missing-at-random assumption, three supplementary sensitivity analyses were performed. First, a complete-case analysis was conducted by restricting the analytic sample only to participants with observed 3-month data (n = 599). Second, an extreme worst-case imputation analysis was conducted by assigning the maximum sample-observed fall count (12 falls), the minimum sample-observed physical function score, and an affirmative ED visit to all 56 intervention dropouts. Third, a tipping-point analysis was implemented for the primary outcome to identify the exact threshold of post-baseline falls among noncompleters required to nullify the statistical significance (p ≥ 0.05) of the intervention effect.

All inferential models were adjusted for the following potential confounders: age, gender (male/female), number of baseline comorbid medical conditions (0, 1–3, 4–6, 7–9), baseline Short FES-I baseline score, baseline STEADI score, baseline fall count within the past year and baseline PFS score (as appropriate to each analysis). Comorbidities considered in analyses included hypertension, coronary heart disease, high cholesterol, asthma, chronic obstructive pulmonary disease/emphysema/chronic bronchitis, chronic kidney disease, prediabetes/borderline diabetes, diabetes and osteoarthritis.

## Results

### Retention & baseline characteristics

**Figure 2.**
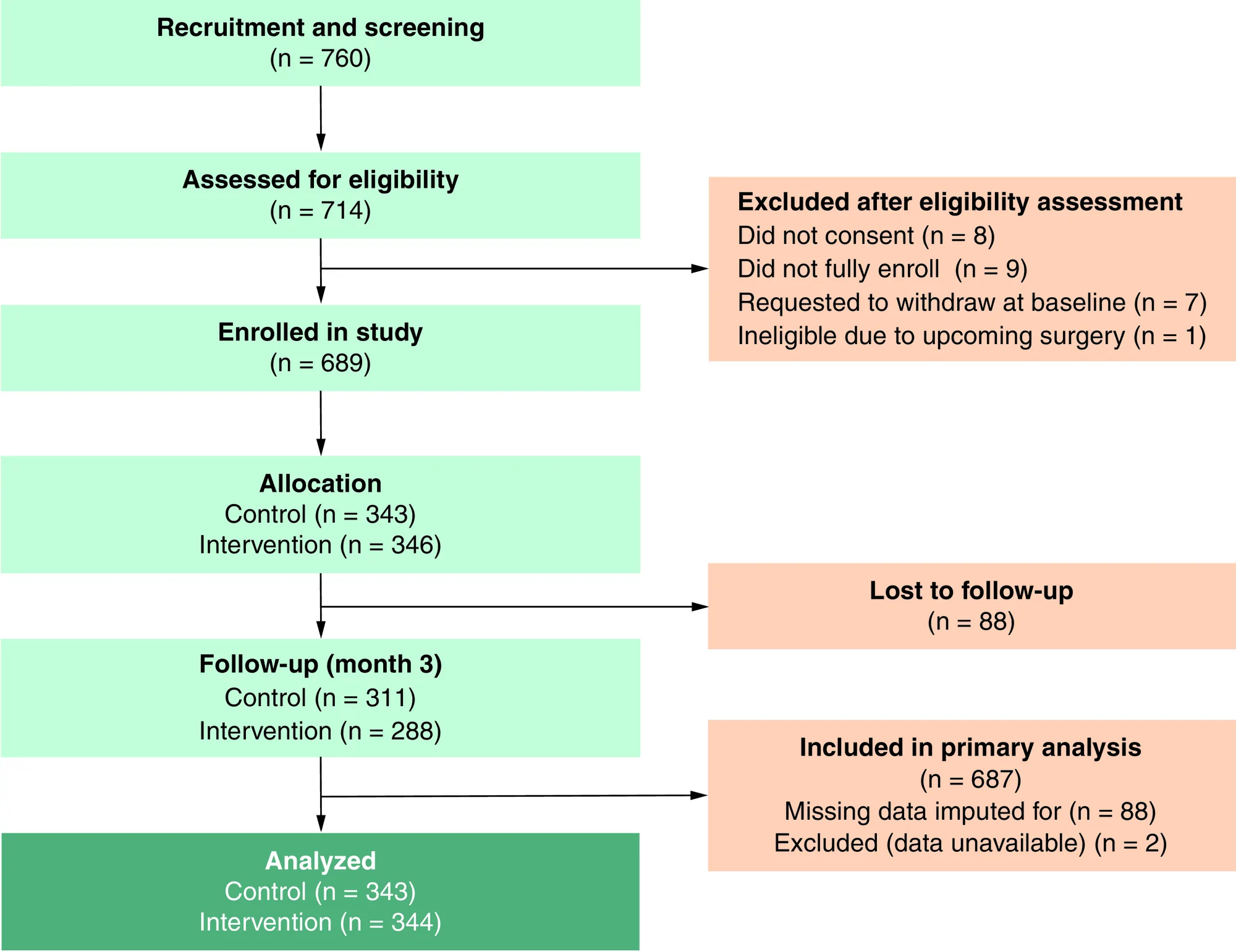
Participant flow diagram.

Of 689 participants enrolled in the study, a total of 687 participants were included in the full analysis set (intervention: n = 344; attention-control: n = 343) after excluding two participants with no post-baseline data. While 599 participants (87.2%) completed the 3-month survey, missing outcome data for the remaining 88 participants were imputed. Overall retention was 87.2%, with 83.7% retention for the intervention group and 90.7% for the attention-control group (Figure 2). No statistically significant difference in attrition was observed between groups. Furthermore, analyses indicated that participants with missing outcome data did not differ significantly from those who completed the study, suggesting that data were missing at random. Baseline demographic characteristics of participants are summarized in Table 1. No statistically significant differences were observed between groups at baseline for demographic or clinical variables, except for a marginal difference in age (attention-control mean: 68.6 years, intervention mean: 69.0 years, p = 0.03). The sample were predominantly female (74.1%), with a mean age of 68.8 years. Most participants identified as White (86.8%), reported at least one comorbidity (91.4%), and rated their health as ‘good’ (44.8%). Importantly, a majority of participants (70.6%) reported experiencing at least one fall in the 12 months prior to enrollment. The average baseline STEADI risk score was 6.6, and the mean Short FES-I was 11.5.

**Table 1.** Baseline demographics (full analysis set).

	Attention-control (N = 343)	Intervention (N = 344)	Total (N = 687)
**Gender, n (%)**			
Female	261 (76.1%)	248 (72.1%)	509 (74.1%)
Male	81 (23.6%)	94 (27.3%)	175 (25.5%)
Transgender	1 (0.3%)	2 (0.6%)	3 (0.4%)
**Age, years, mean (SD)** ^†^	68.6 (2.7)	69.0 (2.8)	68.8 (2.7)
**General health, n (%)**			
Poor	7 (2.0%)	7 (2.0%)	14 (2.0%)
Fair	85 (24.8%)	93 (27.0%)	178 (25.9%)
Good	153 (44.6%)	155 (45.1%)	308 (44.8%)
Very good	82 (23.9%)	80 (23.3%)	162 (23.6%)
Excellent	16 (4.7%)	9 (2.6%)	25 (3.6%)
**Race & ethnicity, n (%)**			
White	296 (86.3%)	300 (87.2%)	596 (86.8%)
Black or African–American	24 (7.0%)	26 (7.6%)	50 (7.3%)
Asian	1 (0.3%)	3 (0.9%)	4 (0.6%)
American Indian or Alaska Native	2 (0.6%)	0 (0%)	2 (0.3%)
Hispanic or Latino/a	6 (1.7%)	4 (1.2%)	10 (1.5%)
Other single and multiple races	14 (4.1%)	11 (3.2%)	25 (3.6%)
**Comorbidities, n (%)**			
None	31 (9.0%)	28 (8.1%)	59 (8.6%)
1–3	226 (65.9%)	227 (66.0%)	453 (65.9%)
4–6	80 (23.3%)	79 (23.0%)	159 (23.1%)
7–9	6 (1.7%)	10 (2.9%)	16 (2.3%)
**Marital status, n (%)**			
Married or living with partner	187 (54.5%)	189 (54.9%)	376 (54.7%)
Widowed, divorced, separated or never married	156 (45.5%)	155 (45.1%)	311 (45.3%)
**Employment status, n (%)**			
Working (for pay, not for pay)	80 (23.3%)	67 (19.5%)	147 (21.4%)
Not working, student, retired or other	263 (76.7%)	277 (80.5%)	540 (78.6%)
**Education, n (%)**			
Less than high school/high school/some college/associate	174 (50.7%)	155 (45.1%)	329 (47.9%)
Bachelor/Master/Doctorate	169 (49.3%)	189 (54.9%)	358 (52.1%)
**Prior fall history (last 12 months)**			
**Reported ≥1 fall, n (%)**	238 (69.4%)	247 (71.8%)	485 (70.6%)
**STEADI, mean (SD)** ^‡^	6.5 (2.2)	6.6 (2.3)	6.6 (2.3)
**Short FES-I, mean (SD)** ^§^	11.4 (3.5)	11.5 (3.9)	11.5 (3.7)

† p < 0.05.

‡ STEADI = Stopping Elderly Accidents, Deaths, and Injuries questionnaire.

§ FES-I = Short Falls Efficacy Scale – International (FES-I) questionnaire.

### Clinical outcomes

Descriptive statistics for clinical outcomes at baseline and 3 months are provided in Table 2. At baseline, there was no significant difference in the reported number of falls within the previous year between groups (p = 0.657). In the primary analysis of all assigned participants, adjusted negative binomial regression revealed a 37% lower fall rate in the intervention group compared with the attention-control group (adjusted IRR: 0.63, 95% CI: 0.48–0.82, p < 0.001; Table 3).

**Table 2.** Unadjusted clinical outcomes at baseline and month 3 (observed data).

Total falls reported, n	Attention-control	Intervention
Baseline^‡^	599	479
Month 3^†^	283	174
**Fall severity, (n) %**		
Baseline^§^		
None	51 (23.6%)	48 (23.5%)
Minor	112 (51.9%)	122 (59.8%)
Moderate	40 (18.5%)	26(12.7%)
Major	13 (6.0%)	8 (3.9%)
Month 3^¶^		
None	49 (37.4%)	41 (45.1%)
Minor	70 (53.4%)	45 (49.5%)
Moderate	10 (7.6%)	4 (4.4%)
Major	2 (1.5%)	1 (1.1%)
**PFS of SF-36, mean (SD)**		
Baseline	59.1 (25.6)	60.0 (25.8)
Month 3^†^	58.6 (25.8)	65.1 (24.8)
**Screened in for low physical functioning, (n) %**		
Baseline	100 (32.2%)	89 (30.9%)
Month 3^†^	108 (34.7%)	75 (26.0%)
**Emergency department (ED) visits, n (%)** ^#^		
Baseline		
Yes	28 (9.0%)	16 (5.6%)
No	283 (91.0%)	272 (94.4%)
Month 3		
Yes	27 (8.7%)	10 (3.5%)
No	284 (91.3%)	278 (96.5%)

† p < 0.05.

‡ Total number of falls reported within the past 1 year.

§ Among participants who reported ≥1 fall within the last year

¶ Among participants who reported ≥1 fall within the last 90 days.

# Participants reporting at least one visit to an emergency department during the 3-month study period.

Values represent observed data from participants who completed the surveys (N = 599; attention-control n = 311, intervention n = 288). Regression models in Tables 3 and 4 utilized the full analysis set (N = 687).

**Table 3.** Negative binomial regression model for fall rates at month 3 (full analysis set).

Variable	Adjusted IRR^†^	95% CI (lower)	95% CI (upper)	p-value
Study group				
Attention-control (N = 343)	Reference	–	–	–
Intervention (N = 344)	0.63	0.48	0.82	<0.001

† An IRR <1.0 indicates a lower rate of falls in the intervention group compared with the attention-control group.

Analyses were performed on the full analysis set (N = 687). Missing data at month 3 were handled using multiple imputation.

Model adjusted for age, gender, number of comorbidities, baseline fall count, baseline Short FES-I score and baseline STEADI score.

IRR: Incidence rate ratio.

No significant differences in worst fall severity were observed between groups at baseline (p = 0.965) or at 3 months (p = 0.961). Ordinal logistic regression indicated no significant difference in fall severity at 3 months between the intervention and attention-control groups (adjusted OR: 1.36, 95% CI: 0.82–2.26, p = 0.227; Table 4).

**Table 4.** Adjusted models for secondary outcomes at month 3 (full analysis set).

Outcome measure	Statistical model	N^†^	Effect estimate^‡^	95% CI (lower)	95% CI (upper)	p-value
Fall severity	Ordinal logistic regression	248	1.36	0.82	2.26	0.227
Physical function (PFS)	Linear mixed-effects model	687	6.86	3.80	9.92	<0.001
ED utilization	Logistic regression	687	0.43	0.24	0.76	0.004

All models were adjusted for age, gender, number of comorbidities, baseline fall count, baseline Short FES-I score and baseline STEADI score.

† Analyses were performed on the full analysis set (N = 687). Missing data at month 3 were handled using multiple imputation. Fall severity analysis includes only participants who reported a fall.

‡ Fall severity and ED utilization estimates represent the adjusted odds ratio (OR) comparing the intervention group to the attention-control group. An adjusted OR <1.0 indicates lower odds of the event (ED visit or higher severity fall) for the intervention group. The physical function estimate represents the β coefficient for the group x time interaction. A positive value indicates greater improvement in the intervention group compared with the control group.

ED: Emergency department; OR: Adjusted odds ratio; PFS: Physical functioning scale (SF-36).

Linear mixed-effects models, which included all available time points for the full analysis set, demonstrated significant improvement in physical functioning in the intervention group compared with the attention-control group over the 3-month period (β = 6.86, p < 0.001; Table 4). Regarding healthcare utilization, the proportion of participants reporting an ED visit remained relatively stable in the attention control group (9.0% at baseline vs 8.7% at month 3) but decreased in the intervention group (5.6% at baseline vs 3.5% at month 3). In the adjusted analysis, intervention group participants had 57% lower odds of ED visits (adjusted OR: 0.43, 95% CI: 0.24–0.76, p = 0.004; Table 4).

### Sensitivity analyses

Three sensitivity analyses evaluated the potential impact of differential attrition (56 intervention noncompleters vs 32 attention control noncompleters). In the complete-case analysis (n = 599), the clinical effectiveness of the intervention remained stable and statistically significant across all outcomes, yielding a 36% lower fall rate (IRR: 0.64, 95% CI: 0.49–0.83, p < 0.001), significant physical function improvements (β = 5.87, 95% CI: 3.45–8.30, p < 0.001) and lower odds of ED visits (OR: 0.37, 95% CI: 0.17–0.76, p = 0.010).

In the extreme worst-case analysis, assigning maximum adverse outcomes to all intervention dropouts caused the primary effect estimates to reverse direction, as anticipated under an unweighted penalty constraint (fall rate IRR: 2.86, 95% CI: 2.12–3.86, p < 0.001; ED utilization OR: 1.81, 95% CI: 1.17–2.81, p = 0.008; though physical function improvements retained statistical significance (β = -4.81, 95% CI: -8.50 to -1.13, p = 0.011).

The tipping point analysis showed that the primary fall rate reduction remained statistically robust until intervention dropouts are assumed to have sustained an average of ≥2 falls during the study period (p = 0.578). This tipping point represents a 3.3-fold increase over the observed mean of 0.6 falls among retained intervention participants, demonstrating that an implausibly high concentration of negative outcomes among dropouts would be required to nullify the primary conclusion (Supplementary Tables 1–3).

### Program engagement

Of the 344 participants in the intervention full analysis set, 56 were lost to follow-up, representing a higher attrition rate compared with the attention-control group (n = 32). Despite this attrition, engagement among retained participants remained substantial; 82.8% of the intervention group completed at least one exercise therapy session. On average, intervention participants completed an average of 25.2 exercise sessions (SD: 24.3) during the 3-month period, corresponding to approximately 2.1 sessions per week. Additional engagement metrics included an average of 12.8 messages initiated to care teams and 20.6 educational articles read per participant over the study period. No program-related injuries or adverse events related to the digital balance program were reported.

## Discussion

This study found a 37% lower fall rate for the intervention group compared with the attention-control group at 3 months. This reduction aligns with the 32–35% effectiveness reported for established, long-term (12-month) in-person interventions like the Otago Exercise Program [9,19], suggesting potential for this compressed 3-month digital delivery to replicate traditional fall prevention modalities to mitigate fall risk in older adults in real-world settings. It is important to note that the primary aim of this study was fall prevention among an at-risk population; consequently, not all participants experienced a fall event during the study period, nor was a history of frequent falling a requirement for participation. The intervention was designed to mitigate risk factors before falls occurred, rather than solely treating those with established fall histories.

While no significant difference in fall severity was observed, this is consistent with broader literature where studies powered for fall rates often lack statistical power to detect changes in less frequent injurious events [20,21].

The intervention group exhibited significant improvements in physical functioning compared with controls. This benefit was particularly pronounced in participants with lower baseline function, highlighting the potential of digital programs to address mobility deficits. The observed high adherence (2.1 sessions/week) aligns with established best practices indicating that optimal fall-reduction outcomes require a cumulative dose of approximately 2 h of balance challenge per week [22,23], providing a mechanism for these clinical gains. Notably, no adverse events were reported in the intervention group, suggesting that digital programs are safe for seniors, including those with lower baseline function. Furthermore, intervention participation was associated with significantly lower odds of ED visits, suggesting potential for reducing costly healthcare utilization [24].

A key strength of this study is its prospective design utilizing a concurrent attention-control group receiving educational content. Another strength was the high engagement rate (82.8% onboarding, an average of 25.2 sessions completed), which contrasts with the high attrition often seen in unguided digital health studies [25]. This supports the value of integrating human health coaching to mitigate dropout. Overall, these findings contribute to the evidence base for scalable, digitally delivered fall prevention strategies that can improve health outcomes and promote healthy aging.

It's important to note that because the attention-control group received passive educational emails, this study design could not isolate the specific clinical efficacy of the exercise therapy component from the nonspecific therapeutic effects of active participation in the digital program. The intervention group engaged with a multi-component program consisting of digital educational modules, a dedicated healthcare team that provided remote behavioral support, progress monitoring, personalized exercise adjustments via in-app messaging and video visits, and automated reminders and engagement prompts from the app to encourage ongoing adherence. These components likely played a meaningful role in fostering participant accountability and compliance. Consequently, the observed 37% reduction in fall rates must be interpreted as the result of the comprehensive digital program, rather than the exercise therapy sessions in isolation.

### Limitations

This study has several limitations. First, the nonrandomized design precludes definitive causal inferences. Additionally, because participants were recruited via separate advertisements, group allocation was subject to self-selection bias and confounding, potentially attracting more highly motivated individuals to the intervention arm. Second, the trial was registered retrospectively; while study outcomes were pre-specified before study inception, retrospective registration limits independent verification of our *a priori* analytical plan and introduces potential risk for selective outcome reporting. Third, while our program engagement metrics stand apart as objective data, reliance on self-reported clinical outcomes without objective physical measures or medical record validation may have introduced a risk of differential reporting bias distinct from generic recall bias. Because participants were unblinded and actively engaged with a clinical care team, intervention participants may have overreported physical function gains due to a Hawthorne effect or underreported falls due to effort justification [26]. Fourth, the intervention group experienced higher attrition than the control group (56 vs 32), likely due to the greater burden of the active program; however, the study was powered *a priori* to account for this, and multiple imputation was utilized to mitigate the potential risk of attrition bias. Fifth, reported reductions in ED visits were not verified by medical records or confirmed as fall-related. Sixth, the 3-month study window limits evaluation of long-term risk reduction and introduces two temporal biases. First, the 12-month baseline versus 3-month follow-up recall mismatch may cause underreporting of baseline events due to memory decay. Second, a short-term window ignores seasonal fluctuations in fall risk. Additionally, concluding data collection immediately at the end of the 3-month intervention precludes assessing whether clinical gains persisted. However, early clinical testing remains a vital early milestone for digital health program iteration and scalability. Seventh, the study population profile introduces generalizability limitations. The study sample, recruited via an online panel, was predominantly white (86.8%), highly educated, and possessed baseline technological literacy and device access. These findings may not be generalizable to more racially, ethnically or socioeconomically diverse older adult populations, or to individuals who are digitally marginalized or lacking reliable internet infrastructure. Finally, unmeasured lifestyle factors (e.g., external exercise habits) were not controlled for. As the program continues to develop, future research will progress to more rigorous randomized designs with longer durations and objective outcome measures.

## Conclusion

Findings suggest that participation in a digitally delivered exercise-based balance program may show preliminary effectiveness in reducing self-reported fall rates, improving physical function scores and lowering patient-reported ED visits compared with an attention-control group. These results highlight the potential value of digital fall prevention interventions and underscore the capacity for high engagement among older adults using connected devices [27]. Digital programs represent an accessible alternative to traditional fall prevention strategies and may play a meaningful role in promoting healthy aging and addressing healthcare utilization in older adults.

## Summary points

- Falls are a leading cause of injury and healthcare costs in older adults, yet barriers such as transportation often limit participation in traditional in-person prevention programs.
- This study evaluated outcomes associated with a digitally delivered balance program featuring app-based personalized exercise therapy, education and care team support (physical therapists and health coaches).
- A nonrandomized controlled trial was conducted with 687 older adults (mean age 68.8 years) at moderate-to-high risk for falls, comparing the digital intervention to an attention-control group.
- In the full analysis set, participation in the digital program was associated with a significantly lower fall rate (IRR: 0.63) compared with the control group at 3 months.
- As an exploratory outcome, participation was associated with significant improvements in physical functioning scores, particularly among those with lower baseline function.
- In an exploratory analysis, participation in the digital program was associated with significantly lower odds of patient-reported emergency department utilization (OR: 0.43) compared with the control group.
- Engagement levels were high, with intervention participants completing an average of 25.2 exercise sessions over 12 weeks (~2.1 sessions/week), a frequency often linked to effective fall prevention outcomes.
- No significant differences were observed in fall severity between groups, a finding consistent with other studies powered primarily to detect differences in fall rates rather than injurious falls.
- These findings suggest that digitally delivered programs may offer a viable alternative to traditional fall prevention strategies for improving health outcomes among older adults with baseline digital literacy.

## Supplementary Material


