# Supplementary material for: Clinical effectiveness of a digitally delivered balance program for older adults: a nonrandomized controlled trial
Source: J Comp Eff Res. 2026 Jul 21;15(9):e260070. doi: 10.57264/cer-2026-0070 (PMC13556878; doi:10.57264/cer-2026-0070)
Supplement: Supplementary file 1 [file cer-15-260070-s1.docx]

**Supplementary Materials**

**Table S1.** Complete-Case Sensitivity Analysis

| **Outcome Measure** | **Statistical Model** | **N** | **Effect Estimate** | **95% CI (Lower)** | **95% CI (Upper)** | ***P* value** |
| --- | --- | --- | --- | --- | --- | --- |
| Fall Rate (Negative Binomial) | Primary Model (Full Analysis Set) | 687 | 0.63 | 0.48 | 0.82 | <.001 |
|  | Complete-Case Sensitivity | 599 | 0.64 | 0.49 | 0.83 | <.001 |
| Physical Function (PFS) (Linear Mixed-Effects Model) | Primary Model (Full Analysis Set) | 687 | 6.86 | 3.80 | 9.92 | <.001 |
|  | Complete-Case Sensitivity | 599 | 5.87 | 3.45 | 8.30 | <.001 |
| ED Utilization (Logistic Regression) | Primary Model (Full Analysis Set) | 687 | 0.43 | 0.24 | 0.76 | 0.004 |
|  | Complete-Case Sensitivity | 599 | 0.37 | 0.17 | 0.76 | 0.010 |

Abbreviations: CI = Confidence Interval; ED = Emergency Department; PFS = Physical Functioning Scale (SF-36).

Effect estimates for Fall Rate represent the Adjusted Incidence Rate Ratio (IRR); estimates for Physical Function represent the β coefficient for the group x time interaction; estimates for ED utilization represent the Adjusted Odds Ratio (OR). Complete-case sample is restricted to participants with observed month 3 data (n=599). Primary models utilize the full analysis set with missing data handles via multiple imputation (n=687). All models are adjusted for age, gender, comorbidities, baseline fall count, baseline short FES-I, and baseline STEADI score.

**Table S2.** Worst-Case Imputation Sensitivity Analysis

| **Outcome Measure** | **Statistical Model** | **N** | **Effect Estimate** | **95% CI (Lower)** | **95% CI (Upper)** | ***P* value** |
| --- | --- | --- | --- | --- | --- | --- |
| Fall Rate (Negative Binomial) | Primary Model (Full Analysis Set) | 687 | 0.63 | 0.48 | 0.82 | <.001 |
|  | Worst-Case Sensitivity Model^1^ | 687 | 2.86 | 2.12 | 3.86 | <.001 |
| Physical Function (PFS) (Linear Mixed-Effects Model) | Primary Model (Full Analysis Set) | 687 | 6.86 | 3.80 | 9.92 | <.001 |
|  | Worst-Case Sensitivity Model^1^ | 687 | -4.81 | -8.50 | -1.13 | 0.011 |
| ED Utilization (Logistic Regression) | Primary Model (Full Analysis Set) | 687 | 0.43 | 0.24 | 0.76 | 0.004 |
|  | Worst-Case Sensitivity Model^1^ | 687 | 1.81 | 1.17 | 2.81 | 0.008 |

Abbreviations: CI = Confidence Interval; ED = Emergency Department; PFS = Physical Functioning Scale (SF-36).

Worst-case analysis assigns the maximum sample-observed fall count (12 falls) to all 56 intervention dropouts for the fall rate model, the minimum sample-observed PFS score for the physical function model, and an affirmative ED visit (Yes) for the healthcare utilization model. All models are adjusted for age, gender, comorbidities, baseline fall count, baseline short FES-I, and baseline STEADI score.

^1^The effect estimate reversed direction under this extreme parameter penalty.

**Table S3.** Tipping-Point Analysis for Primary Outcome (Fall Rate)

| **Falls Assigned to Intervention Dropouts** | **Adjusted IRR** | **95% CI (Lower)** | **95% CI (Upper)** | ***P* value** | **Model Context/Note** |
| --- | --- | --- | --- | --- | --- |
| 0 | 0.55 | – | – | <.001 | Assumes zero falls among non-completers. |
| Base Model (MICE) | 0.63 | 0.48 | 0.82 | <.001 | Primary Analysis (Observed Completer Mean = 0.6) |
| 1 | 0.75 | – | – | 0.015 | Assumes dropouts out-fell completers. |
| 2 | 0.94 | – | – | 0.578 | Tipping Point (Intervention effect nullified; *p* ≥ 0.05) |
| 3 | 1.13 | – | – | 0.311 | Direction of effect begins to invert. |
| 4 | 1.32 | – | – | 0.024 | Statistically significant in the opposite direction. |
| 6 | 1.71 | – | – | <.001 | Extreme deviation from observed data. |
| 12 | 2.86 | 2.12 | 3.86 | <.001 | Worst-case Scenario (sample maximum assigned). |

Abbreviations: IRR = Incidence Rate Ratio; CI = Confidence Interval; MICE = Multiple Imputation by Chained Equations.

The primary fall rate IRR is derived from a negative binomial regression adjusted for all baseline covariate. Retained intervention participants who completed the study averaged 0.6 falls (174 total ÷ 288 participants). The tipping point of ≥ 2 assigned falls represents an implausible 3.3-fold increase over the actual observed fall rate of program completers.
